# Disease-Associated Mutations Prevent GPR56-Collagen III Interaction

**DOI:** 10.1371/journal.pone.0029818

**Published:** 2012-01-04

**Authors:** Rong Luo, Zhaohui Jin, Yiyu Deng, Natalie Strokes, Xianhua Piao

**Affiliations:** Division of Newborn Medicine, Department of Medicine, Children's Hospital and Harvard Medical School, Boston, Massachusetts, United States of America; Medical College of Georgia, United States of America

## Abstract

GPR56 is a member of the adhesion G protein-coupled receptor (GPCR) family. Mutations in GPR56 cause a devastating human brain malformation called bilateral frontoparietal polymicrogyria (BFPP). Using the N-terminal fragment of GPR56 (GPR56^N^) as a probe, we have recently demonstrated that collagen III is the ligand of GPR56 in the developing brain. In this report, we discover a new functional domain in GPR56^N^, the ligand binding domain. This domain contains four disease-associated mutations and two N-glycosylation sites. Our study reveals that although glycosylation is not required for ligand binding, each of the four disease-associated mutations completely abolish the ligand binding ability of GPR56. Our data indicates that these four single missense mutations cause BFPP mostly by abolishing the ability of GPR56 to bind to its ligand, collagen III, in addition to affecting GPR56 protein surface expression as previously shown.

## Introduction

Adhesion GPCRs are a relatively new family of GPCRs that have a very long N-terminal extracellular domain. In humans, there are a total of 33 members of adhesion GPCRs that are thought to mediate cell-cell and cell-extracellular matrix interaction, with GPR56 as the first one linked to a human developmental malformation [1], [2]. Mutations in *GPR56* cause BFPP, a specific human brain malformation [3], [4]. To date, a total of fourteen BFPP-associated mutations have been identified, including one deletion, two splicing, and eleven missense mutations [2], [5]. Based on the fact that all of the missense mutations render an identical clinical phenotype as the deletion mutation, it is presumed that all missense mutations result in a null allele [4]. Previously, we have shown that all of the missense mutations affect GPR56 protein trafficking and cell surface expression to variable degrees [6]. However, we can only say for certain that the two mutations in the GPCR proteolytic site (GPS) domain, C346S and W349S, cause a brain malformation through trapping the mutated proteins in the endoplasmic reticulum [6].

Most recently, we have discovered that the ligand of GPR56 is collagen III [7]. In this report, we aim to identify and characterize the binding domain of GPR56 to collagen III. Although the N-terminal extracellular domain of GPR56, GPR56^N^, is heavily glycosylated, we provide strong evidence that glycosylation is not required for ligand binding. Moreover, disease-associated mutations in the ligand binding domain completely eliminate its ligand binding ability, revealing the molecular mechanism for GPR56-related brain malformation.

## Results

### GPR56^N^ binds specifically to collagen III in the developing brain

**Figure 1:**
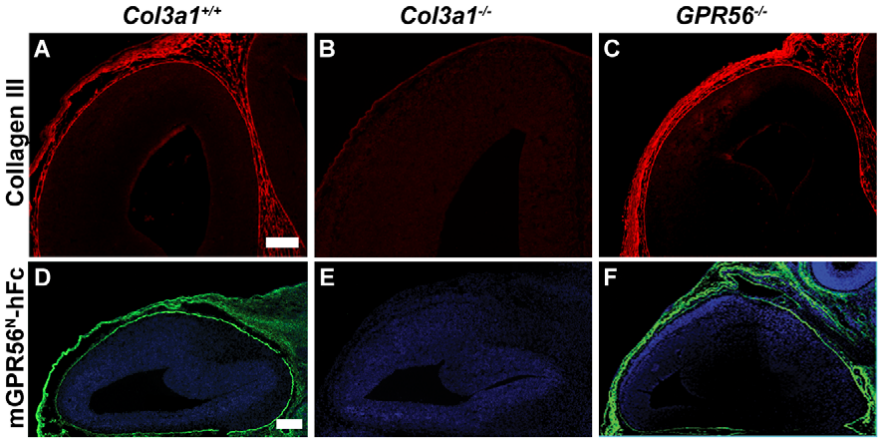
GPR56^N^ binds specifically to collagen III in the developing brain. (**A–C**) Immunostaining of collagen III in E14.5 mouse brain sections of wild type, *Col3a1^−/−^*, and *Gpr56^−/−^* revealed an expression of collagen III (red) in the meninges and pial basement membrane of wild type and *Gpr56^−/−^* brains. Deletion of *Col3a1* completely abolished the expression of collagen III. Scale bar, 100 µm. (**D–F**) Strong GPR56^N^ binding was detected in meninges and pial basement membrane of wild type and *Gpr56^−/−^*, but not *Col3a1^−/−^* mouse brains. Nuclear counterstain was performed with Hoechst 33342 (blue). Scale bar, 200 µm.

We have previously shown that GPR56^N^ binds a putative ligand in the meninges and pial basement membrane and subsequently demonstrated that the ligand of GPR56 is collagen III in the developing brain [7], [8]. To examine the binding specificity of GPR56^N^ to collagen III, we performed a putative ligand binding assay in wild type, *Col3a1* ^−/−^, and *Gpr56* ^−/−^ brains. Both *Col3a1* ^−/−^ and *Gpr56* ^−/−^ mice are true null allele mutants (Fig. 1B) [8]. Deletion of *Gpr56* has no effect on the expression of collagen III protein and GPR56^N^ binding patterns (Fig. 1C and F), whereas loss of *Col3a1* completely removed the binding of GPR56^N^ (Fig. 1E). Taken together, our data supports that GPR56^N^ binds specifically to collagen III in the developing brain.

### The ligand binding domain of GPR56 is within aa 27–160

**Figure 2:**
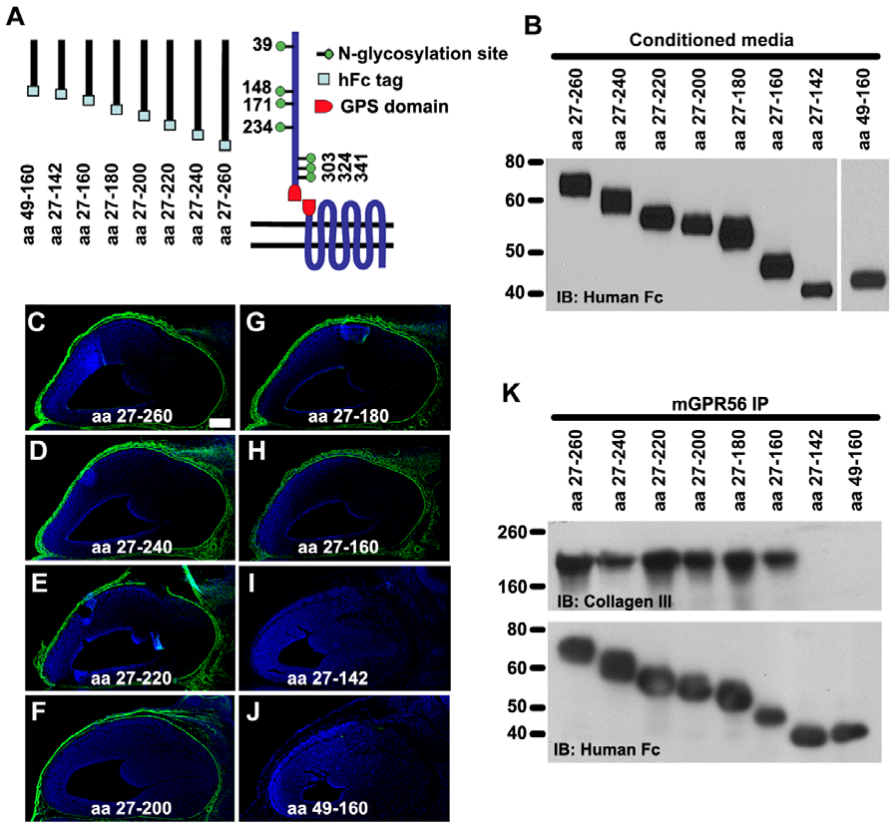
The ligand binding domain of GPR56. (**A**) The truncated GPR56^N^-hFc fusion protein constructs are schematically shown, as well as the full length GPR56 protein with its identified N-glycosylation sites. (**B**) The fusion constructs were transfected into HEK-293T cells. Secreted proteins in the conditioned media were collected, concentrated, and verified by western blot. (**C–J**) Putative ligand binding on E14.5 mouse cortex. The shortest fragment with a specific binding pattern (green) is the truncated GPR56^N^-hFc fusion protein containing aa 27–160 (H). Nuclear counterstain was performed by Hoechst 33342 (blue). Scale bar, 200 µm. (**K**) The binding of collagen III and various truncated GPR56^N^-hFc was confirmed by co-IP. Anti-hFc immunoblot served as a loading control.

We have previously shown that the deletion of aa 93–143 completely abolished the ligand binding ability of GPR56, suggesting that the ligand binding domain lies in the most N-terminal region of GPR56^N^ [8]. To identify the ligand binding domain of GPR56, we engineered a series of truncated GPR56^N^-hFc fusion protein constructs by gradually trimming GPR56^N^ from both the N- and C-terminus (Fig. 2A). The constructs were transiently expressed in HEK-293T cells to generate fusion proteins that were naturally glycosylated (Fig. 2B). The concentrated fusion proteins were then used for ligand binding assays on embryonic day (E) 14.5 mouse cortices. We were able to observe putative ligand binding with all of the fusion proteins except the ones containing aa 27–142 and aa 49–160 (Fig. 2C–J). The shortest fragment that has full ligand binding capacity was the fusion protein containing aa 27–160 (Fig. 2H).

To further verify the putative ligand binding data, we carried out a series of co-immunoprecipitation (co-IP) experiments using meningeal fibroblasts as the ligand source. We failed to detect collagen III in the co-IP protein complexes of either aa 27–142 or aa 49–160, despite comparable fusion protein levels (Fig. 2K). Altogether, our data supports the conclusion that the fragment of aa 27–160 contains the ligand binding domain of GPR56.

### N-glycosylation is not required for ligand binding

**Figure 3:**
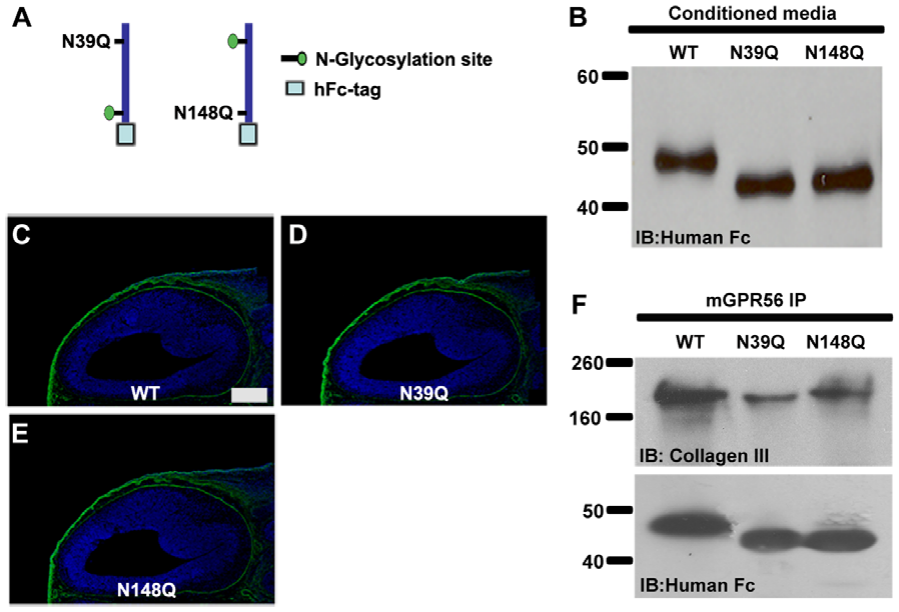
Glycosylation mutations of GPR56 did not affect its ligand binding. (**A**) The schematic representation of two separate N-glycosylation mutations within the GPR56 ligand binding domain is shown. (**B**) Secreted fusion proteins in the conditioned media were collected, concentrated, and verified by western blot. (**C–E**) Putative ligand binding on E14.5 mouse cortex. The N-glycosylation mutation did not impair GPR56 ligand binding (green). Nuclear counterstain was performed by Hoechst 33342 (blue). Scale bar, 200 µm. (**F**) The binding ability of the two N-glycosylation mutants to collagen III was confirmed by co-IP. Anti-hFc immunoblot served as a loading control.

The ligand binding domain of GPR56 contains two N-glycosylation sites, N39 and N148. To investigate whether glycosylation at each of these two sites is required for ligand binding, we generated two mutations, N39Q and N148Q, in the fragment of aa 27–160 to individually remove the corresponding N-glycosylation (Fig. 3A). The mutant proteins were detected in the conditioned media of transiently transfected cells and, as expected, both mutations abolished one N-glycosylation site resulting in a protein that migrates faster on SDS-PAGE (Fig. 3B). The mutant fusion proteins were concentrated from the conditioned media and used for both the putative ligand binding assay and co-IP experiments. Both mutants retained a ligand binding capacity comparable to the wild-type protein, suggesting glycosylation of GPR56 is not necessary for ligand binding (Fig. 3C–F).

### Disease-associated mutations abolish ligand binding

**Figure 4:**
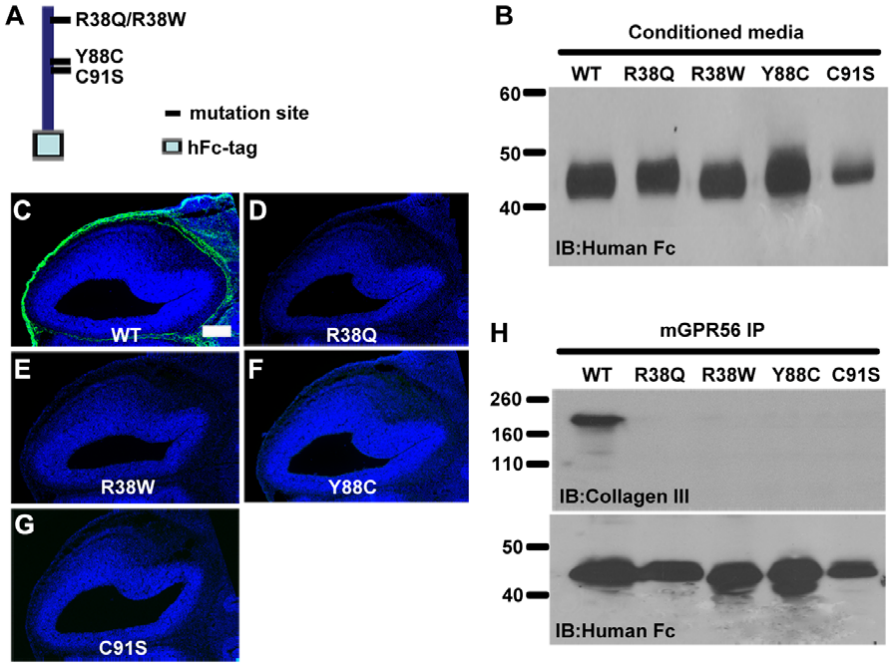
Disease-associated GPR56 abolished its ligand binding. (**A**) GPR56^N^-hFc aa 27–160 schematic. The positions of the disease-associated mutations within the ligand binding domain are shown. (**B**) Secreted fusion proteins in the conditioned media were collected, concentrated, and verified by a western blot. (**C–G**) Putative ligand binding on E14.5 mouse cortex. Each of the four disease-associated mutations completely killed the receptor-ligand binding ability in contrast to the strong binding signal (green) displayed by the wild type fusion protein (**C**). Nuclear counterstain was performed with Hoechst 33342 (blue). Scale bar, 200 µm. (**H**) The impaired binding of disease-associated mutants to collagen III was confirmed by co-IP. Anti-hFc immunoblot served as a loading control.

The four reported disease-associated mutations in the ligand binding domain are R38Q, R38W, Y88C, and C91S. We have previously shown that these four mutations decreased mutant proteins expression on the cell surface and its secretion into the conditioned media [6]. It is not clear, however, how each of the four missense mutations completely demolishes the receptor function. To study whether these mutations also affect ligand binding, we generated individual mutant ligand binding domains by site-directed mutagenesis in the pFUSE-hFc2 construct containing aa 27–160 of GPR56 (Fig. 4A). We were able to detect the fusion mutant proteins in the conditioned media of the transfected cells (Fig. 4B). The mutant fusion proteins were concentrated from the conditioned media and used for the putative ligand binding assay and co-IP experiments. None of these four mutant proteins could bind the ligand in both the putative ligand binding assay and co-IP experiments, propositioning that the four missense mutations completely killed the ligand binding ability of GPR56 (Fig. 4C–H).

### Disease-associated mutations fail to rescue collagen III-mediated neural migration inhibition

**Figure 5:**
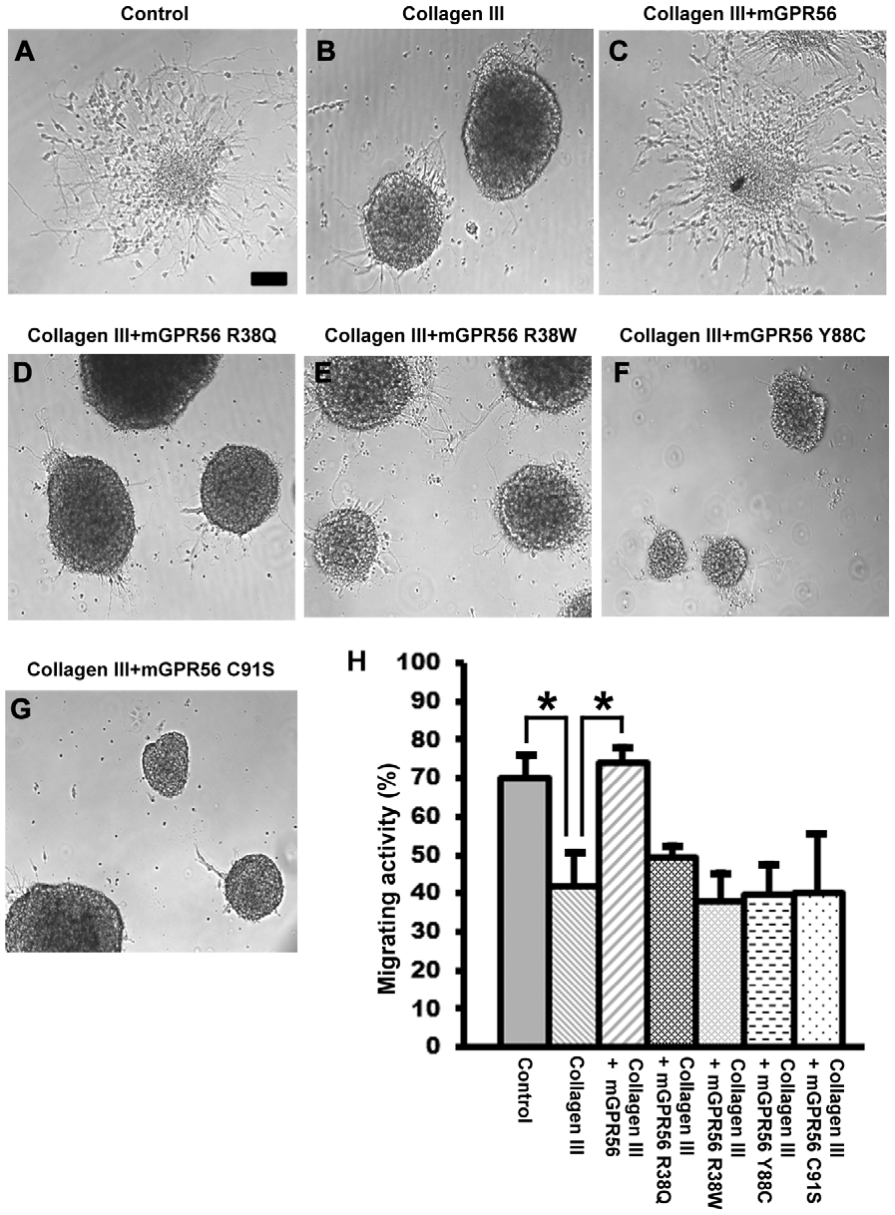
Mutant mouse GPR56 proteins failed to rescue collagen III-mediated neural migration inhibition. (**A–G**) Neurospheres were generated and plated on PDL-coated dishes in regular neurosphere culture medium. After 20 h, the cultures were changed to experimental medium containing collagen III (84 nM) with or without wild type or mutant GPR56 proteins (90 nM), or carrier solution (acetic acid). Representative images are shown. Scale bar, 100 µm. (**H**) The degree of collagen III-mediated migration inhibition was quantified as a percentage of the migrating neurospheres. Data are presented as mean ± SD, n = 3 for each group. *P<0.01, Student t test.

We have thus far demonstrated a loss of interaction between mutant GPR56 proteins and collagen III by tissue binding and protein pull down assays. However, the functional implication of this observation is not clear. We have previously shown that collagen III inhibits neural migration via GPR56 and that wild type GPR56^N^ can rescue this inhibition [7]. To further investigate the impact of BFPP-associated mutations on the receptor function, we performed a neurosphere migration assay to examine whether the four BFPP-associated mutations - R38Q, R38W, Y88C, and C91S - functionally abolish receptor-ligand interaction. As expected, in contrast to the wild type GPR56^N^, none of the four mutant proteins were able to reverse the collagen III-mediated neural migration inhibition (Fig. 5).

### Wild type human GPR56^N^ binds to both human and mouse collagen III, whereas mutant GPR56 proteins lose their ligand binding ability

**Figure 6:**
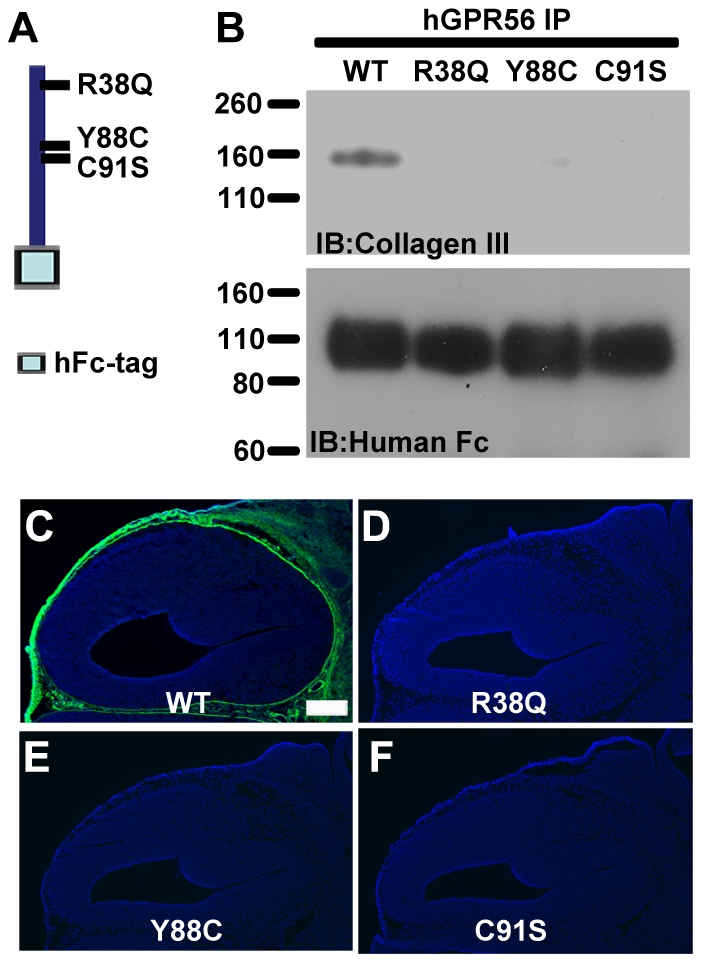
Human GPR56 binds to both mouse and human collagen III and disease-associated GPR56 mutations abolish their ligand binding ability. (**A**) Human GPR56^N^-hFc aa 27–382 schematic. The positions of the disease-associated mutations are shown. (**B**) Co-IP of human GPR56^N^ and purified human collagen III. Collagen III was detected in GPR56 IP complex, but not in mutant GPR56 complexes. Anti-hFc immunoblot served as a loading control. (**C–F**) Putative ligand binding of human GPR56^N^-hFc on E14.5 mouse cortex. Strong binding signal was detected (green) with wild type human GPR56^N^-hFc protein staining, whereas a loss of signal occurred in mutant proteins. Nuclear counterstain was performed with Hoechst 33342 (blue). Scale bar, 200 µm.

We have thus demonstrated that mouse GPR56 binds to both mouse and human collagen III. To study whether the interaction can extend to the human GPR56 protein, we constructed a human GPR56^N^-hFc fusion protein (hGPR56^N^-hFc) and the corresponding BFPP-associated mutants: R38Q, Y88C, and C91S. We first performed co-IP experiments using purified human collagen III. As expected, we can only detect collagen III in wild type hGPR56^N^ but not in the mutant hGPR56^N^ co-IP protein complexes (Fig. 6B). We next examined the tissue binding activity on E14.5 mouse brain sections. As illustrated in Figure 6 C–F, wild type hGPR56^N^ binds to mouse collagen III whereas mutant hGPR56^N^ fails to exhibit any binding activity on mouse embryonic brains. Taken together, our data suggests that there is no species restriction between mouse and human in regards to the interaction of GPR56 and collagen III.

## Discussion

GPR56 is a particularly distinct member of the adhesion GPCRs since its mutations are associated with a devastating human brain malformation called BFPP [3], [9], [10]. There are a total of eleven reported null allele missense mutations associated with BFPP, two of which reside in the GPS domain and cause brain malformation by demolishing the autoproteolytic process, thus trapping the mutant proteins in the endoplasmic reticulum [5], [10]. A recent study by Lin's group indicates that mutations in the tip of GPR56^N^ likely render the receptor null status by affecting their binding to a putative cellular ligand in HT1080 cells [11]. Here, we show four mutations in the tip of GPR56^N^ impair the receptor function by aborting its ligand binding ability, in addition to affecting the receptor cell surface expression.

GPR56 is cleaved at aa 382 via a GPS-mediated autoproteolytic process [6], [12], [13]. Although the cleaved GPR56^N^ lacks a transmembrane sequence, it is expressed on the cell surface via an unknown mechanism [6]. In addition, GPR56^N^ is secreted into the conditioned media [6], [13]. We previously showed that the four missense mutations on the tip of GPR56^N^ (R38Q, R38W, Y88C, and C91S) affect the secretion of GPR56^N^ into the conditioned media [6]. In this study, we were able to detect secreted mutant proteins in their short forms. The possible explanations could be either that the pFUSE-hFc2 vector was more efficient in driving protein expression/secretion since it uses the IL2 signal peptide sequence or that replacing aa 160–382 with the large hFc fragment facilitated the shedding of the fusion protein.

GPR56 functions in a capacity to mediate cell-extracellular matrix interaction. Our recent finding demonstrates that collagen III is the ligand of GPR56 in the developing brain [7]. Collagen III is a major collagen in the connective tissues with integrin α1β1 and α2β1 both serving as it's receptors [14], [15]. In this report, we identified the binding domain of GPR56 to collagen III. Since there is no consensus amino acid sequence between integrin α1β1/α2β1 and GPR56, it is likely that GPR56 binds to a different region of collagen III.

In addition to its vital function in brain development, GPR56 also plays an important role in tumor growth and metastasis. GPR56 was originally cloned by two independent groups in 1999, one using a degenerative PCR approach and the other through differential display in high and low metastatic melanoma cell lines [16], [17]. The latter group revealed that GPR56 is significantly down regulated in high metastatic melanoma cell lines, indicating a possible role of GPR56 in tumor metastasis [17]. It was subsequently demonstrated that GPR56 inhibits tumor growth/metastasis, likely by regulating VEGF production and tumor angiogenesis [13], [18]. In addition, they showed that GPR56 binds to TG2, a major crosslinking enzyme in the extracellular matrix, at aa 108–177 [13], [18]. Although knockdown of TG2 did not lead to an increase of VEGF, deleting the TG2 binding domain led to enhanced angiogenesis and tumor growth [18]. There is some overlap between the collagen III and TG2 binding domains on GPR56. It is highly desirable to reveal the crystal structure of these two binding domains in order to fully understand how GPR56 dynamically interacts with both collagen III and TG2 in both the developing brain as well as tumor growth and metastasis.

## Materials and Methods

### Ethics Statement

Experiments were performed in accordance with National Institutes of Health guidelines for the care and use of laboratory animals, and with the approval of the Animal Care and Use Committee of Children's Hospital Boston.

### Mice

*Col3a1* knockout mice were obtained from the Jackson Laboratory with the strain name C.129S4 (B6)-Col3a1tm1Jae/J in a BALB/c background. The *Gpr56* knockout mice, kindly provided by Genentech, were produced in collaboration between Genentech and Lexicon Genetics to analyze the function of ∼500 secreted and transmembrane proteins. All animals were treated according to the guidelines of the Animal Care and Use Committee of Children's Hospital Boston (approval ID: A3303-01).

### Histology and Immunohistochemistry

Histology analysis was carried out as previously described [7], [8]. Frozen sections were collected on a cryostat. Sections were incubated with rabbit anti-human collagen III antibody (Lifespan Biosciences). Primary antibodies were visualized by appropriate fluorophore-conjugated secondary antibodies. Images were captured using a Nikon 80i upright microscope. Representative photographs were obtained with the same exposure setting for both the control and mutant.

### Plasmid Constructions

The human IgG Fc-tagged mouse GPR56^N^ (mGPR56^N^-hFc) and its truncated fragments were constructed in pcDNA3.1-zeo vector (Invitrogen). The hFc tag was cloned into the Xho I/Apa I sites of the vector. Mouse GPR56^N^ and its truncated fragments, including the GPR56 signal peptide sequence, aa 1–26, were generated by PCR using primers listed in Table S1 and subsequently fused to hFc by inserting into the Nhe I/Xho I sites.

Our previous data showed that BFPP-associated mutations affect protein intracellular trafficking and secretion [6]. To increase protein secretion, we cloned the mouse GPR56 ligand binding domain, aa 27–160, into the pFUSE-hFc2 vector (Invivogen) that contains the IL2 signal peptide sequence. N-glycosylation mutations and BFPP-associated mutations were created by site-directed mutagenesis using the QuikChange II XL Site-Directed Mutagenesis kit (Stratagene), as previously described [6]. The primers used for the site-directed mutagenesis are listed in Table S2.

The human IgG Fc-tagged human GPR56^N^ (hGPR56^N^-hFc) was also constructed in pFUSE-hFc2 vector (Invivogen) between Nco1 and BglII sites. The primers used for human GPR56^N^ PCR are listed in Table S3. BFPP-associated mutations were created by the same approach described above using the primers listed in Table S4.

### Generation of hFc fusion protein and putative ligand binding assay

Each of the above GPR56^N^-hFc expression constructs was transiently transfected into HEK-293T cells (obtained from ATCC). The culture media was changed to serum-reduced OPTI-MEM 24 hours after transfection. The conditioned media was harvested 48–72 hours later, and concentrated as previously described [8]. For the neurosphere migration assay, the mouse GPR56 27–160 hFc wild type and mutant proteins were purified through protein A column (GE Healthcare).

Time pregnant wild type mice in the CD-1 background were ordered from Charles River. Embryonic day (E) 14.5 wild type mouse brain sections were obtained at 12 µm in thickness on a cryostat (Leica). Equivalent amounts of fusion proteins were used as probes to examine their binding ability to mouse brain sections. The localization of GPR56^N^-hFc proteins were visualized by fluorescein-conjugated rabbit anti-human IgG antibody (Thermo Scientific).

### Co-immunoprecipitation (co-IP) and western blot analysis

Co-IP for mouse GPR56^N^ and collagen III and immunoblotting were done as previously described, using meningeal fibroblasts as the ligand source [7]. Purified human collagen III (Abcam) was used in co-IP to test the interaction of human GPR56^N^ and collagen III. Protein-G beads were used to pull-down the GPR56^N^-hFc protein complex. Immunocomplexes were subjected to SDS-PAGE and western blot using rabbit anti-human collagen III antibody (Lifespan Biosciences), and rabbit anti-human IgG Fc antibody (Thermo Scientific) following standard protocols.

### Migration assay

Neurosphere generation and migration assay were done as described previously [7]. The neurospheres were plated in a 48-well dish precoated with 100 µg/ml poly-D-lysine (PDL) and cultured in neuron culture medium overnight. After the neurospheres adhered to PDL substrate, the culture medium was then replaced with one of the experimental mediums: neuron culture medium with 84 nM purified human collagen III (Abcam) with or without 90 nM wild type or mutant GPR56^N^, or with carrier solution (acetic acid) as control. The neurospheres were imaged and the number of migrating neurospheres was quantified after a 2 day culture. It is worth noting that Abcam only guarantees the quality of purified or recombinant human collagen III by western blot analysis. We prescreened the biological function of each lot by a neural migration assay prior to a bulk order, since there is a significant variation between each lot.

## Supporting Information

Table S1 Primers for mouse truncated GPR56^N^-hFc cloning.
(DOC)

Table S2 Primers for mouse GPR56^N^-hFc site-directed mutagenesis.
(DOC)

Table S3 Primers for human GPR56^N^-hFc cloning.
(DOC)

Table S4 Primers for human GPR56^N^-hFc site-directed mutagenesis.
(DOC)
