# Supplementary material for: Disease-Associated Mutations Prevent GPR56-Collagen III Interaction
Source: PLoS One. 2012 Jan 4;7(1):e29818. doi: 10.1371/journal.pone.0029818 (PMC3251603; doi:10.1371/journal.pone.0029818)
Supplement: Table S1 — Primers for mouse truncated GPR56N-hFc cloning. (DOC) [file pone.0029818.s001.doc]

**Table S1. Primers for mouse truncated GPR56^N^-hFc cloning.**

Shared forward 5’-CCGCTGGCTAGCATGGCTGTCCAGGTGCTGCGGCAG-3’

27-260 reverse 5’-CTCCACCTCGAGACTCTGCTCCTCCTCCTTCTGGGAGTG-3’

27-240 reverse 5’-CTCCACCTCGAGCAGCTTCCACACTGTAGCATTGACCC-3’

27-220 reverse 5’-CTCCACCTCGAGGCTCACAGAGGTCAGCTTTGACTCCA-3’

27-200 reverse 5’-CTCCACCTCGAGGGGCCGCTTGGCAGCCTTTTGAGGGT-3’

27-180 reverse 5’-CTCCACCTCGAGCTTGAGATCACACATGTCCACAGATG-3’

27-160 reverse 5’-CTCCACCTCGAGGGAGAAGATGAAGCTCGGAGCCCCAG-3’

27-142 reverse 5’-CTCCACAGATCTGGAGCTGACAGAGGTGGCGATCAGCGG-3’

49-160 forward 5’-GATATCGGCCATGGATCAATCTTCAGAGCCTCACATCTTTG-3’

49-160 reverse 5’-CTCCACAGATCTGGAGAAGATGAAGCTCGGAGCCCCAGGC-3’
