# Supplementary material for: Disease-Associated Mutations Prevent GPR56-Collagen III Interaction
Source: PLoS One. 2012 Jan 4;7(1):e29818. doi: 10.1371/journal.pone.0029818 (PMC3251603; doi:10.1371/journal.pone.0029818)
Supplement: Table S2 — Primers for mouse GPR56N-hFc site-directed mutagenesis. (DOC) [file pone.0029818.s002.doc]

**Table S2. Primers for mouse GPR56^N^-hFc site-directed mutagenesis.**

N39Q Forward: 5’-GCTTCTGTGGCCAGCGGCAACAGACCCAACAGAGCACCC -3’

Reverse: 5’-GGGTGCTCTGTTGGGTCTGTTGCCGCTGGCCACAGAAGC-3’

N148Q Forward: 5’- CCTGGCAGATTCCCCAGCAAACCAGCCTGCCTGGGGC -3’

Reverse: 5’- GCCCCAGGCAGGCTGGTTTGCTGGGGAATCTGCCAGG -3’

R38Q Forward: 5’- CCGCTTCTGTGGCCAGCAGAACCAGACCC -3’

Reverse: 5’- GGGTCTGGTTCTGCTGGCCACAGAAGCGG -3’

R38W Forward: 5’- CCGCTTCTGTGGCCAGTGGAACCAGACCC -3’

Reverse: 5’- GGGTCTGGTTCCACTGGCCACAGAAGCGG -3’

Y88C Forward: 5’- CCTAGAGGGCTCTGTCACTTCTGCCTCTAC -3’

Reverse: 5’- GTAGAGGCAGAAGTGACAGAGCCCTCTAGG -3’

C91S Forward: 5’- GGCTCTATCACTTCTCCCTCTACTGGAGTCGCC -3’

Reverse: 5’- GGCGACTCCAGTAGAGGGAGAAGTGATAGAGCC -3’
