# Supplementary material for: Disease-Associated Mutations Prevent GPR56-Collagen III Interaction
Source: PLoS One. 2012 Jan 4;7(1):e29818. doi: 10.1371/journal.pone.0029818 (PMC3251603; doi:10.1371/journal.pone.0029818)
Supplement: Table S3 — Primers for human GPR56N-hFc cloning. (DOC) [file pone.0029818.s003.doc]

**Table S3. Primers for human GPR56^N^-hFc cloning.**

Forward (with Nco1 site) 5’- GGCCATGGAAGACTTTCGCTTCTGCAGCC -3’

Reverse (with BglII site) 5’- GTTAGATCTCAAGTGGT TGCAGAAGCAGG -3’
