# Supplementary material for: Disease-Associated Mutations Prevent GPR56-Collagen III Interaction
Source: PLoS One. 2012 Jan 4;7(1):e29818. doi: 10.1371/journal.pone.0029818 (PMC3251603; doi:10.1371/journal.pone.0029818)
Supplement: Table S4 — Primers for human GPR56N-hFc site-directed mutagenesis. (DOC) [file pone.0029818.s004.doc]

**Table S4. Primers for human GPR56^N^-hFc site-directed mutagenesis.**

R38Q Forward: 5’- GCTTCTGCAGCCAGCAGAACCAGACACACAGG -3’

Reverse: 5’- CCTGTGTGTCTGGTTCTGCTGGCTGCAGAAGC -3’

Y88C Forward: 5’- CAGGGGCCTCTGCCACTTCTGCCTCTACTGG -3’

Reverse: 5’- CCAGTAGAGGCAGAAGTGGCAGAGGCCCCTG -3’

C91S Forward: 5’- GCCTCTACCACTTCTCCCTCTACTGGAACCGAC -3’

Reverse: 5’- GTCGGTTCCAGTAGAGGGAGAAGTGGTAGAGGC -3’
